# A Method to Study Honey Bee Foraging Regulatory Molecules at Different Times During Foraging

**DOI:** 10.3389/finsc.2021.723297

**Published:** 2021-09-22

**Authors:** Asem Surindro Singh, Machathoibi Chanu Takhellambam

**Affiliations:** ^1^Department of Neuroscience, National Centre for Biological Sciences, Tata Institute of Fundamental Research (TIFR), Bangalore, India; ^2^Department of Biotechnology, Manipur University, Imphal, India

**Keywords:** honey bee foraging, learning and memory, immediate early genes expression, *Egr-1* (early growth response protein 1), *Hr-38*, *Kakusei*

## Abstract

The foraging of honey bees is one of the most well-organized and admirable behaviors that exist among social insects. In behavioral studies, these beautiful insects have been extensively used for understanding time–space learning, landmark use, and the concept of learning. Highly organized behaviors such as social interaction and communication are systematically well-organized behavioral components of honey bee foraging. Over the last two decades, understanding the regulatory mechanisms underlying honey bee foraging at the cellular and molecular levels has been increasingly interested to several researchers. Upon the search of regulatory genes of brain and behavior, immediate early (IE) genes are considered as a good tool to begin the search investigation. Our two recent studies have demonstrated three IE genes, namely, *Egr-1, Hr38*, and *Kakusei*, playing a role in the daily foraging of bees and their association with learning and memory during foraging. These studies further evidence that IE genes can be used as a tool in finding the specific molecular/cellular players of foraging in honey bees and its behavioral components such as learning, memory, social interaction, and social communication. In this article, we provide the details of the method of sample collection at different times during foraging to investigate the foraging regulatory molecules.

## Introduction

Honey bee foraging consists of several behavioral components that include search of food, identification and memorization of food source location, carrying and storing of food, and interaction and communication (1–6). Inside the artificial bee house, honey bees flew several times to and fro carrying pollen/nectar from the feeder to the hive. All these behaviors can be observed by feeding the bees few meters away from the hive inside the bee house. Generally, cap honey bees or European honey bee *Apis mellifera* is widely used for understanding behavioral dynamics of social insects. Though it looks small and tiny, this insect displays incredible means to communicate each other. During foraging, honey bees of the same colony share information and communicate each other through a typical movement called “waggle dance” of honey bees that looks like the numerical figure eight. For the first time, this waggle dance of bees was translated by Austrian ethologist Karl Ritter von Frisch, and he received the Nobel Prize in Physiology or Medicine in 1973 for his incredible effort toward investigating the sensory perceptions in honey bees (7–10).

So far, the foraging behavior of honey bees has been extensively studied and available data are plenty. This has opened incredible research opportunities for understanding the regulatory mechanisms in various ways in honey bees. However, the reports on the underlying mechanisms in the brain are very limited. In recent years, there is increasing research interest in this direction. Understanding cellular and molecular regulators of honey bee foraging not only would be confined within insects, but also can offer some guidelines/clues toward unwinding the complexity of neural circuitry systems and molecular underpinnings in higher animals and humans (11). Because humans and other large animals have many similar behavioral features with honey bees, there is sequence homology in the genes across various species.

Upon the search of foraging regulatory genes in the brain, to begin with IE genes is a promising way of investigation because they are well-known neural markers. The IE genes had been found to have persisting roles from the first stages of brain development into adulthood, showing possible inherent features in everyday brain activity (12). It also showed to have dramatic roles in phenotypic changes that occurred in neurons (13). There are many IE gene encoded transcription factors that are rapidly induced within the neurons, and some were delayed (14, 15) in response to different stimuli and cellular environments that result in neuronal capacities with short- and long-lasting phenotypic changes (12, 16). Following the stimulation, early response neurons react from milliseconds to minutes, which involve first and second messenger systems and phosphatases, whereas late response persists from hours to days and even to permanent changes coupled with gene expression changes (16, 17). The late response neurons were found to be linked to learning, memory, sensitization processes, and drug tolerance habits (16, 17). Remarkably, during the process of nerve stimulation, the IE genes have been noted as first activated genes that link to membrane events and nucleus (18). Therefore, regulation in the IE gene expression level is considered to be the first part in the general neuronal response to a natural stimulus. Thus, it is a promising way to start with IE genes in the planning of experimental strategies from scratch toward finding specific molecular and neuronal pathway that link to a specific behavior.

In our two recent studies, we have found the involvement of three IE genes, *Egr-1, Hr-38*, and *Kakusei*, in the daily foraging of honey bees as well as their possible role in learning and memory processing during foraging (4, 5). Moreover, the possible role of downstream genes of *Egr-1*, ecdysone receptor (*EcR*), dopamine/ecdysteroid receptor (*DopEcR*), dopamine decarboxylase (*Ddc*), and dopamine receptor 2 (*Dr2*) were also revealed. Subsequently, involvement of *Hr38, EcR*, and *DopEcR*, which are part of the ecdysteroid signaling pathway, in learning and memory processes, has also been indicated (4). In these studies, we have performed sample collections/behavioral experiments while the bees were on the process of foraging, and such an experiment was not reported before. Another interesting part of the study is that the behavioral experiments were performed in a more or less natural environment or may be considered as a semi-natural condition. The bee house is airy and the bees could fly back and forth and continued to forage without any disturbance or a negligible disturbance. In this article, we focus to highlight the method of sample collection in particular and the molecular experiments in detail that had been published briefly in our two previous articles (4, 5) with few additional data.

## Materials and Methods

### Behavioral Experiment and Sample Collection

European honey bee species (also known as cap honey bees) *Apis mellifera* colonies were purchased from the local bee keepers in Bangalore, Karnataka, India. The bee colonies were placed inside the bee house of the institute, National Center for Biological Sciences (NCBS), Tata Institute of Fundamental Research (TIFR), Bangalore, India. *A. mellifera* is one of the most common and widely domesticated honey bee species in the world. Therefore, this honey bee species is not endangered or threatened and available in almost every place in the world. Behavioral test was performed inside the bee house of NCBS, which is an outdoor flight cage. The bee house is 12 m in length, 5 m in height, and 2.5 m in width. The bees were fed with pollen in a green plastic plate and 1 M sucrose solution in a yellow plastic plate. The distance of feeders from the beehive was 10 m, and the two feeders were kept 1.5 m apart from each other. The bees were fed every day from 14:00 to 17:00 h.

Sample collection was started after the foraging bees had learned and already adapted about the location of feeders and had visited the feeders for several days. The collected samples were subjected to gene expression profiling, and for this, only nectar foragers were collected. The procedures were briefly described in our two previous articles (4, 5), and in this article, we described the method in detail.

### Sample Collection Procedure and Grouping

#### Sample Collection During Foraging

For the 0-min group sample, the first arriving foragers at the feeder plate were caught before presenting the sucrose solution on the plate and the caught bees were immediately flash frozen in liquid nitrogen. For catching the bees, 50-ml falcon tubes with tiny pores were used. As soon as the first collection was over, sucrose solution was poured on the plate, and some of the first arriving foragers were gently marked using Uni POSCA Paint Markers (Uni Mitsubishi Pencil, UK) on the head while they are drinking sucrose solution and time count was immediately started. The marked bees that arrived on the feeder during their repeated trips were gently caught at a series of different time points with 15-min intervals up to 2 h. The time points were 15, 30, 45, 60, 75, 90, 105, and 120 min. After catching, the bees were immediately flash frozen in liquid nitrogen. About 24 bees were collected in a day from 14:00 h to 16:00 h, i.e., one to two bees for each time point and continued in the same manner in the following days until 5 bees were obtained for each time point group. The collected samples were stored at −80°C for further experiment.

#### Sample Collection for Before and After Foraging Groups

For collecting the before-foraging samples, some first arriving bees on the feeder at 14:00 h were paint-marked (in the same way as above) and collected in the following morning at 09:00 h inside the hive, before they started flying out from the hive for foraging. In the case of after-foraging samples, the bees that were paint-marked were caught in the hive in the evening at 18:00 h of the same day of paint marking after the bees finished foraging. Gentle care had been taken always during the collection procedure in order not to disturb the bees' normal behavior and to avoid inducing stress phenomena; in this way, minimal interactions between the collector and the bees could be accounted (19–21). The caught bees were immediately flash frozen in liquid nitrogen and stored at −80°C for further processing. In this case, five bees in each group were collected at the same time on the same day of collection.

#### Sample Collection for Food Unrewarded Group

This group consisted of only the foraging bees collected on the empty feeder plate, and the collection was done for 1 h at four time points with intervals of 15 min. The 0-min samples were collected at 14:00 h on the empty feeder plate and immediately followed by paint marking of some bees (in the same way as above) for the collection in the four subsequent time points. Since bees had to be collected without food reward in the remaining four time points, a simple trick was applied. A small amount of 1 M sucrose solution was presented on the plate to let the bees continue foraging on the feeder but a little portion was allowed to be accessible to the bees for drinking by covering the sucrose solution with a transparent bowl. This way, the marked bees were stopped from drinking sucrose solution and assured that they did not touch the sucrose solution while some unmarked bees were allowed to drink. The paint-marked bees were caught as soon as they landed on the feeder plate. About one to two bees were collected in each day and the collection was done from 14:00 to 16:00 h, and each group had five bees at least. The bees were immediately flash frozen in liquid nitrogen as soon as they were caught and stored at −80°C for further experiments.

### Gene Expression Profiling

#### Brain Dissection

The frozen bees at −80°C were removed and lyophilized at −50°C with vacuum at 0.420 mBar for 20 min, using a lyophilizer (Freeze Zone1 PlusTM 4.5 L cascade Freeze Dry System, Labconco Corporation, Kanas City). The bee head was placed in a glass chamber containing 100% ethanol placed on a dry ice platform and the brain dissection was performed under a light microscope. Soon after the dissection, the whole brain was immediately placed into a 1.5-ml Eppendorf tube placed on dry ice, and 500 μl of Trizol (Trizol Reagent, Ambion RNA, Life Technology) was added. Thus, a prepared sample was ready for total RNA preparation. The same procedure was followed for every bee brain dissection.

#### RNA Preparation and cDNA Conversion

The frozen sample was thawed after placing on ice and the brain was homogenized using an electronic homogenizer (Micro-Grinder Pestle Mixer, RPI Research Products International) with pestle (Micro-Tube Sample Pestles, Research Products International). By centrifugation at 10,000 g for 5 min at 4°C, the total RNA, protein, DNA, and cell debris fractions were separated. The upper clear fraction containing RNA was removed gently, leaving the lower portion containing genomic DNA, tissue debris, and the protein fractions. Thus, the total RNA was extracted. Then, cDNA was prepared from the total RNA, by using the kits supplied by Invitrogen (Thermo Fisher Scientific). The manufacturer's protocol was followed in cDNA preparation.

#### Quantitative Real-Time PCR

The prepared cDNA from each brain as in the above was subjected to qPCR using a 7900HT Fast Real Time PCR System (Applied Biosystem, Singapore). The qPCR reaction mixture for each sample was prepared in 200-μl microcentrifuge tubes with 10-μl reaction volume that contained cDNA, oligonucleotide primers (Sigma-Aldrich) specific to target genes, and SYBR Green [KAPA Syber1 FAST PCR Master Mix (2X) ABI Prism1]. The qPCR cycles followed Applied Biosystem protocol. *Rp49* was used as endogenous control in every qPCR run. The details of the target genes and the oligonucleotide primers are provided in Table 1.

**Table 1:** Gene locations, oligonucleotide primers, and the qPCR product amplicon sizes.

**Gene name**	**NCBI gene ID**	**Chromosome no. and location**	**Oligonucleotide primer sequence 5′-3′**	**Amplicon size**
*Egr-1*	726302	LG15	F-GCTCTGAGGGTGATTTCTCG	138 bp
		NC_007084.3	R-GAGAAACCGTTCTGCTGTGA	
*Hr38*	551592	LG13	F-GCACGAATCAATCTTCTACAACC	108 bp
		NC_007082.3	R-AATCCGCCAGGGTACTACATC	
*Kakusei*	100049563	LG2	F-TGGGTAGGGTTGGTAAGGGAA	91 bp
		NC_007071.3	R-ACACGAAACCATCCTGCCAC	
*Erk7*	408917	LG4	F-ACCCGGTCCGAAGAAGAAAT	67 bp
		NC_007080.3	R-CAGGCCAAAAGTCTGAGAATCA	
*c-Jun*	726289	LG9	F-CCCTTCAGCAATTTAACCTTATC	78 bp
		NC_007078.3	R-CGTGGCGGCATCCAAA	
*GluR*	411220	LG7	F-GGGATCGCCTCATATACCCA	71 bp
		NC_007076.3	R-GAGCGAACCAAAGGCTGTTT	
*5-Ht_2_α*	411323	LG9	F-GTCTCCAGCTCGATCACGGT	126 bp
		NC_007078.3	R-GGGTATGTAGAAGGCGATCAGAGA	
*Rp49*	406099	LG11	F-CAGTTGGCAACATATGACGAG	124 bp
		NC_007080.3	R-AAAGAGAAACTGGCGTAAACC	
*DopR*	406111	LG15	F-ACAGAATTCCGAGAAGCGTTCA	79 bp
		NC_007084.3	R-ATTCGCTAGTCGACGGTTCATTT	

*LG, linkage group*.

**Figure 1:**
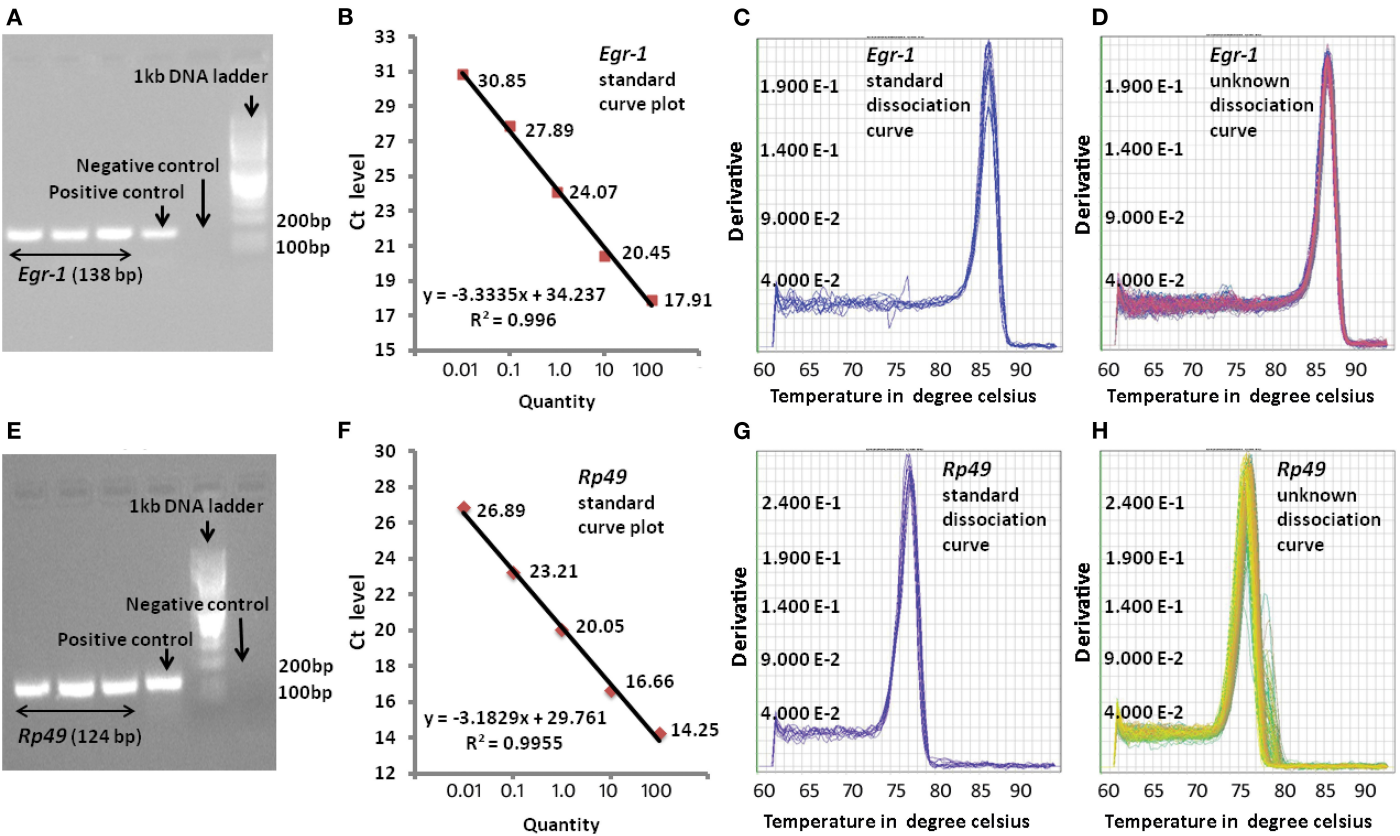
Representative pictures of qPCR analysis. **(A)** qPCR product gel electrophoresis for *Egr-1* on 2% agarose gel, **(B)** standard curve for *Egr-1* with *R*^2^ = 0.0993, **(C)** dissociation curve for *Egr-1* standard cDNA amplification, **(D)** dissociation curve for *Egr-1* unknown cDNA amplification, **(E)** qPCR product gel electrophoresis for *Rp49* on 2% agarose gel, **(F)** standard curve for *Rp49* with *R*^2^ = 0.994, **(G)** dissociation curve for *RP49* standard cDNA amplification, and **(H)** dissociation curve for *Rp49* unknown cDNA amplification.

In order to examine the efficiency of qPCR amplification of the target genes, a positive and negative control were also used in every qPCR run. In case of positive control, instead of cDNA, 20 times diluted genomic DNA prepared from the whole honey bee [using the method by (22)] was added, whereas for negative control, neither genomic DNA nor cDNA was added in the PCR reaction mixture. The rest of the PCR master mix components in both the positive and negative control was the same as that of the target genes. For standard curve, cDNA was used as template with five different concentrations that were serially diluted by 10-fold. The amplification of target genes by qPCR was confirmed by gel electrophoresis in 2% agarose gel (Figure 1). Amplification of single amplicon was also examined from the machine-generated melt curve or dissociation curve. We used the relative standard curve method for relative gene expression analysis. The standard curve with a correlation coefficient (*R*^2^) value >0.98 and close to 1 was considered for further analysis (Figure 1). The CT values thus generated were used for the relative gene expression analysis.

### Statistical Analysis

The relative gene expression level was calculated using the relative standard curve method with the help of SDS 2.4 software provided with the 7900HT Fast Real system. The standard deviation was calculated following Applied Biosystem's “Guide to performing relative quantification of gene expression using real-time quantitative PCR.” The fold changes at all the time points were determined relative to time t0, and the statistical significance was examined using one-way ANOVA with Tukey–Kramer *post-hoc* multiple comparison test and the analysis was carried out with the help of GraphPad InStat software (23). Normal distribution of each comparing group was tested using the D'Agostino & Pearson omnibus normality test.

## Results

### Expression Analysis for Target Genes by qPCR

The expression profiles for all the target genes were measured with the help of qPCR. Highly efficient qPCR results were considered for the relative gene expression analysis. Efficiency of each qPCR result was investigated at different stages before analyzing the relative gene expression levels of target gene with respect to the house keeping gene *Rp49*. Gel electrophoresis of qPCR products were performed for confirming the specific amplification of the target genes, without/negligible primer dimer formation and free of non-specific gene amplification. Standard curve plots with correlation coefficient (*R*^2^) value >0.98 and close to 1 were taken as considerably accurate in the relative measurement of the unknown amount of expression of the target genes. The dissociation curves or melting curves were further examined for the specific amplification of the target genes in each cycle of the qPCR. A representative picture of efficiency checking is shown in Figure 1.

### IE Genes, *Egr-1, Hr-38*, and *Kakusei* Expression During Foraging, Before Foraging, and After Foraging

**Figure 2:**
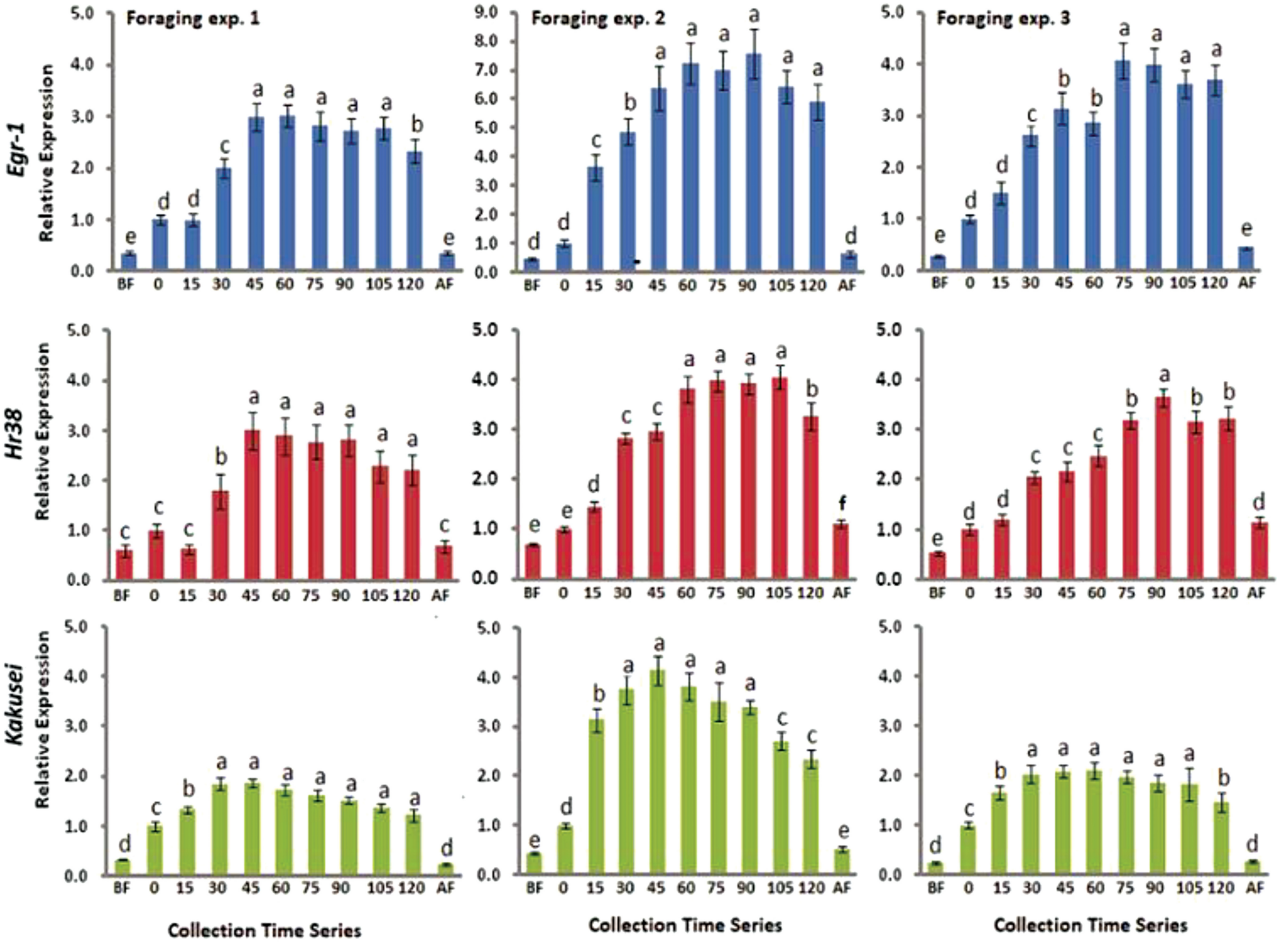
Bar graphs for three IE gene expression during, before, and after foraging. Blue, red, and green represent *Egr-1, Hr38*, and *Kakusei* expression during foraging at a known feeder. Data are shown as fold changes with respect to 0 min (mean value was set as 1), which indicates the presentation of the feeder and start of continuous foraging. BF, before foraging and AF, after foraging. Foraging experiments 1, 2, and 3 represent three independent replicate experiments. Statistical significance was calculated using one-way ANOVA with Tukey-Kramer *post-hoc* multiple comparison tests and significant levels were represented by *p*-values. *p* ≥ 0.05 is considered to have no significant difference and *p* < 0.05 is considered to be significantly different between the means. The statistical difference between the adjacent groups is represented by letters a, b, c. Same letters above the adjacent error bars represent no significant difference and different letters represent a significant difference. Sample size for each time point is *n* = 5.

**Figure 3:**
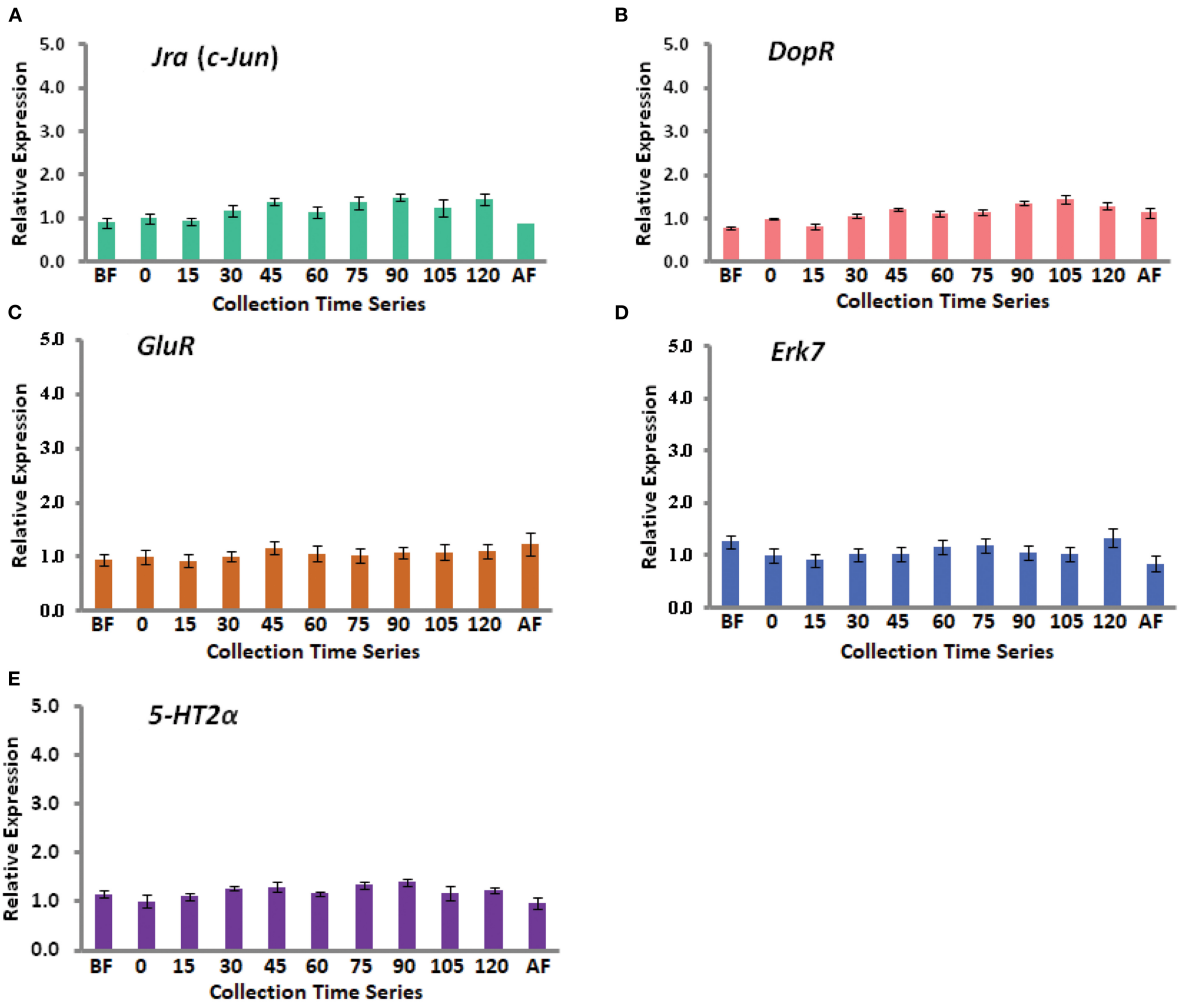
Gene expression profile for the five insignificant genes. **(A)** With green bars, **(B)** with red bars, **(C)** with brown bars, **(D)** with blue bars, and **(E)** with violet bars represent *Jra* (*c-Jun*), *DopR, GluR, Erk7*, and *5-HT2*α expression during the food reward foraging. The fold changes differences were measured with respect to 0 min (mean value was set as 1 at this time point). Each time point has a sample size of *n* = 5.

In this study, we have combined most of our recent published data in two different journals (4, 5). A total of nine genes have been considered including a house keeping gene *Rp49*. The eight genes are *Egr-1, Hr38, Kakusei, c-Jun (Jra), Erk7, GluR, 5-Ht2*α, and *DopR*, and further details of these genes are provided in Table 1. Among the four IE genes, *Egr-1, Hr38, Kakusei*, and *c-Jun (Jra)*, we observed that *Egr-1, Hr38*, and *Kakusei* were found to have significant transient overexpression during the reward foraging within 2 h. The results are summarized in Figure 2 and further details of the statistical significance are provided in Table 2. The other four genes *Erk7, GluR, 5-Ht2*α, and *DopR* as well as *c-Jun (Jra)* were found to have no statistical difference in their expression during foraging as shown in Figure 3. Furthermore, there is no expression change in case of before and after foraging groups except for *Hr38* exp. 2 (*p* < 0.05).

**Table 2:** Statistical significance analysis for *Egr-1, Hr38*, and *Kakusei* at different points in 2 h of sample collection during food reward foraging, after foraging, and before foraging.

**Time points** ***p*****-value**	**Exp. 1** ***p*****-value**	**Exp. 2** ***p*****-value** ***p*****-value**	**Exp. 3** ***p*****-value** ***p*****-value**	**Exp. 1** ***p*****-value** ***p*****-value**	**Exp. 2** ***p*****-value** ***p*****-value**	**Exp. 3 *p*-value** ***p*****-value**	**Exp. 1** ***p*****-value** ***p*****-value**	**Exp. 2** ***p*****-value** ***p*****-value**	**Exp. 3** ***p*****-value** ***p*****-value**
	***Egr-1***			***Hr38***			***Kakusei***		
BF−0	<0.001	ns	<0.01	ns	ns	<0.01	<0.001	<0.01	<0.001
0–15	ns	<0.001	ns	ns	<0.01	ns	<0.001	<0.001	<0.001
15–30	<0.001	<0.05	<0.001	<0.001	<0.001	<0.001	<0.001	<0.01	<0.05
30–45	<0.001	<0.01	<0.05	<0.001	ns	ns	ns	ns	ns
45–60	ns	ns	ns	ns	<0.001	ns	ns	ns	ns
60–75	ns	ns	<0.001	ns	ns	<0.001	ns	ns	ns
75–90	ns	ns	ns	ns	ns	<0.01	ns	ns	ns
90–105	ns	ns	ns	ns	ns	<0.01	ns	>0.001	ns
105–120	<0.05	ns	ns	ns	>0.001	ns	ns	ns	<0.05
120–AF	<0.001	<0.001	<0.001	<0.001	>0.001	<0.001	<0.001	>0.001	<0.001

*NB, Statistics were performed using one-way ANOVA with Tukey–Kramer multiple comparison test; before foraging is abbreviated as BF; after foraging is abbreviated as AF; time points 0–120 are in minutes. p < 0.05 are considered significant and multiple comparison post-hoc test was performed to control the chances of resulting increase of statistical significance due to running many tests simultaneously. “ns” stands for not significant (p > 0.05)*.

### *Egr-1, Hr-38*, and *Kakusei* Expression During Foraging at the Extended Hour

**Figure 4:**
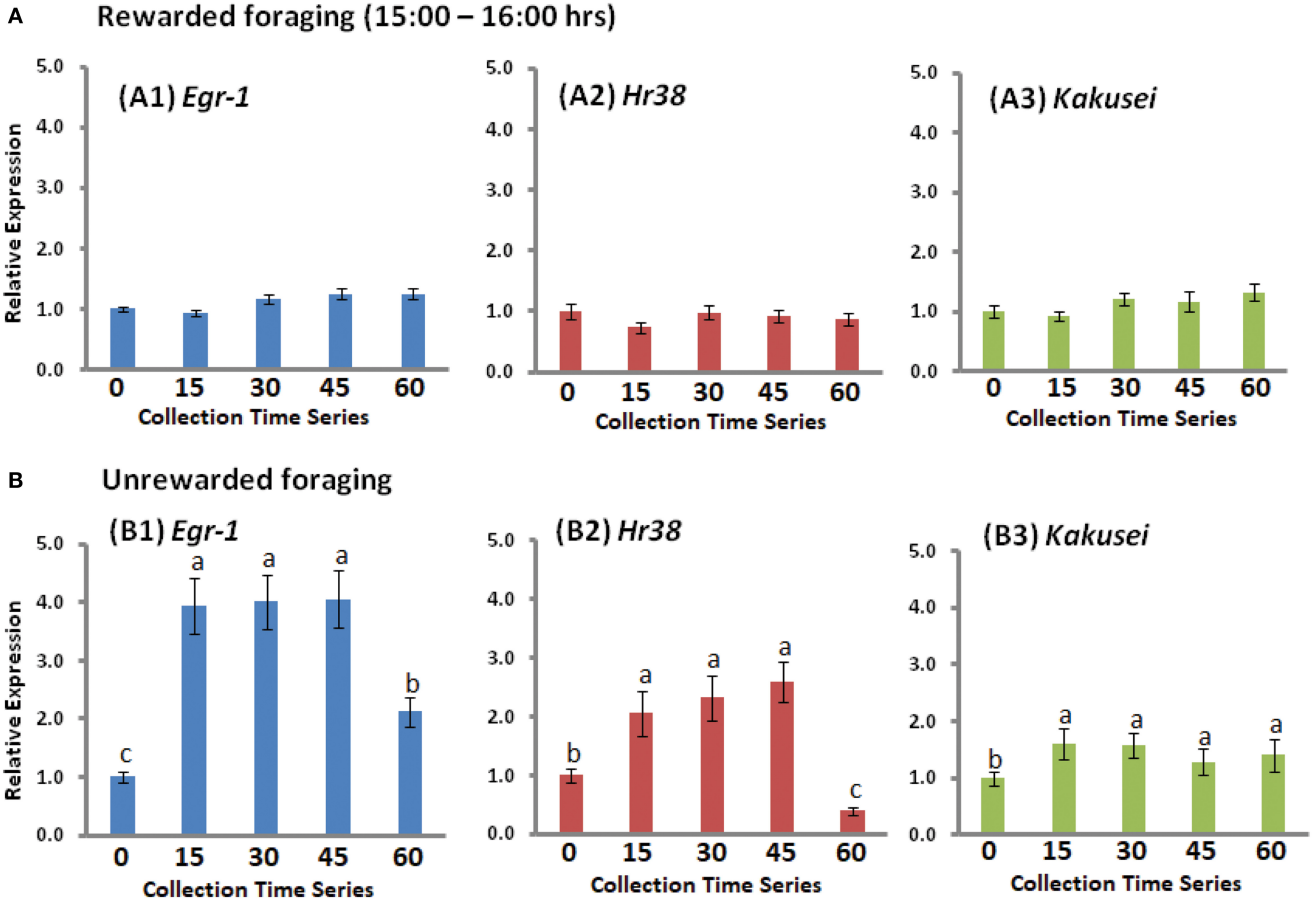
**(A)** The expression profile of *Egr-1* (blue), *Hr38* (red), and *Kakusei* (green) during the extended collection hour (15:00 h−16:00 h). **(B)** The expression of *Egr-1* (blue), *Hr38* (red), and *Kakusei* (green) during unrewarded foraging. The fold change differences were calculated with respect to 0 min (mean value was set as 1 at this time point). Each time point has a sample size of *n* = 5. For statistics, one-way ANOVA with Tukey–Kramer *post-hoc* multiple comparison test was performed. *p* ≥ 0.05 is considered to have no significant difference and *p* < 0.05 is considered to be significantly different between the means. The statistical difference between the adjacent groups is represented by letters a, b, or c. Same letters above the adjacent error bars represent no significant difference and different letters represent a significant difference. Sample size for each time point is *n* = 5.

In another experiment, we collected the samples for 1 h with 15-min intervals, which is after 2 h of reward foraging, from 16:00 h to 17:00 h, during which bees were continuing feeding. These data have not been published before. It may be noted that the bees had been fed since 14:00 h, as everyday routine. This experiment was conducted because we further wanted to check if there might be any change in the gene expression during the later hour of foraging. We did not find any change in gene expression of all the three genes *Egr-1, Hr38*, and *Kakusei*, as shown in Figure 4A. This shows that the transient overexpression occurred only within the first 2 h of reward foraging.

### IE Genes, *Egr-1, Hr-38*, and *Kakusei* Expression Level During Unrewarded Foraging

In case of unrewarded foraging, the three IE genes *Egr-1, Hr38*, and *Kakusei* were increased in the first 15 min of foraging upon the presentation of empty feeder plate and no further increase thereafter, as the food was not rewarded to the bees. The statistical significance between the 0-min and 15-min group for the three genes are presented with *p* < 0.001 for *Egr-1/Hr38* and *p* < 0.01 for *Kakusei*. Subsequently, between 45 min and 60 min, the significant difference for *Egr-1* and *Hr38* is leveled with *p* < 0.001 and not significant for *Kakusei*. Gene expression level began to reduce at about 45 min. The summary of this result for *Egr-1, Hr38*, and *Kakusei* is figuratively presented in Figure 4B.

## Discussion

Our recent publications have supported that IE genes can be used as tools in searching for cellular and molecular mechanisms underlying the foraging behavior of honey bees (3–5, 24). In those studies, we investigated the role of IE gene involvement during the daily foraging and with the examination of reasonably longer duration, which was for 2 h, during the foraging. However, we have not published in detail about the method we used, basically about sample collection at different time points in 2 h during the daily rewarded foraging of bees. Here, we report the detailed method with the addition of a small amount of unpublished data, shown in Figure 4A.

All these data clearly show that the three IE genes, *Egr-1, Hr38*, and *Kakusei*, were transiently expressed during the first 2 h of rewarded foraging and after which the three genes have no significant role, as indicated by their expression levels that continued to decline, even though the bees continued foraging with food reward. This indicates the presence of active roles of the downstream genes of these IE genes in the subsequent hours of foraging. From these results, one may have an idea of choosing an appropriate time during foraging to test other IE genes as well as their upstream or downstream molecular players and designed further experiments for finding specific roles they play in the specific behavioral features of honey bees during foraging. Our findings also have indicated the possible role of those three IE genes in learning and memory processing and associative learning. Further research is needed in order to find the specific molecular pathways underlying those behaviors. The advantage of working with this method is that the behavioral experiments were performed in more or less natural conditions as the bees continued foraging without showing any visible disturbance at a stretch of 2 h or more of sample collection. Therefore, we assumed that little inconvenience caused by paint marking to the bees is negligible. These results also display to choose a specific time point for sample collection during rewarded/unrewarded foraging in the further studies of *Egr-1, Hr38*, and *Kakusei* associated genes. It would be more interesting if we could categorically study the specific age group of the foraging bees in case there may be variations in the gene expression level among the different age groups of foraging bees and, moreover, examine the overexpression of the target proteins that could further validate our data to the next level; these are the limitations of this study.

In other reports, Beckmann and Wilce (18) mentioned that IE gene encoded proteins can be individually regulated in different regions of the brain depending on the type of the stimuli. This suggests that the same/different IE gene expression at different parts of the brain induced by different stimuli may signal to perform different behavioral tasks; in other words, different behaviors correspond to IE gene expression at different parts of the brain depending on the type of the stimulus. The IE genes also rapidly and transiently induced within minutes of stimulation in the absence of *de novo* protein synthesis, and regulation of IE gene production is necessary for the cells, because in turn it can activate the downstream target molecules that typically function as a part of a network of constitutively expressed proteins (25). Furthermore, different IE genes reach their peak levels at different times even though they expressed immediately after the stimulation (26, 27). Our data agree with the report, as in the case of three genes we investigated, *Kakusei* reached its peak earlier than *Egr-1* and *Hr-38*. These several lines of evidences clearly reveal that it is a good choice to start with IE genes when we need to start from scratch for unwinding the molecular and cellular mechanisms underlying specific behaviors of honey bees during foraging, such as learning, memory, social interaction, and social communication.

## Data Availability Statement

The datasets presented in this study can be found in online repositories. The names of the repository/repositories and accession number(s) can be found in the article/supplementary material.

## Author Contributions

All authors listed have made a substantial, direct and intellectual contribution to the work, and approved it for publication.

## Funding

This work was completed with the help of Research Associate Fellowship provided by Council of Scientific and Industrial Research, Government of India, Award No. 09/860(0167)/2015—EMR-1 and Bridging Postdoctoral Fellowship by the National Center for Biological Sciences, TIFR, India, to AS.

## Conflict of Interest

The authors declare that the research was conducted in the absence of any commercial or financial relationships that could be construed as a potential conflict of interest.

## Publisher's Note

All claims expressed in this article are solely those of the authors and do not necessarily represent those of their affiliated organizations, or those of the publisher, the editors and the reviewers. Any product that may be evaluated in this article, or claim that may be made by its manufacturer, is not guaranteed or endorsed by the publisher.
